# Predicting Redox Conditions in Groundwater at a National Scale Using Random Forest Classification

**DOI:** 10.1021/acs.est.3c07576

**Published:** 2024-03-07

**Authors:** Anthony J. Tesoriero, Susan A. Wherry, Danielle I. Dupuy, Tyler D. Johnson

**Affiliations:** † U.S. Geological Survey, 601 SW Second Avenue, Suite 1950, Portland, Oregon 97204, United States; ‡ U.S. Geological Survey, 6000 J Street, Placer Hall, Sacramento, California 95819, United States; § U.S. Geological Survey, 4165 Spruance Road, Suite 200, San Diego, California 92101, United States

**Keywords:** redox reactions, machine learning, dissolved oxygen, nitrate, manganese, groundwater

## Abstract

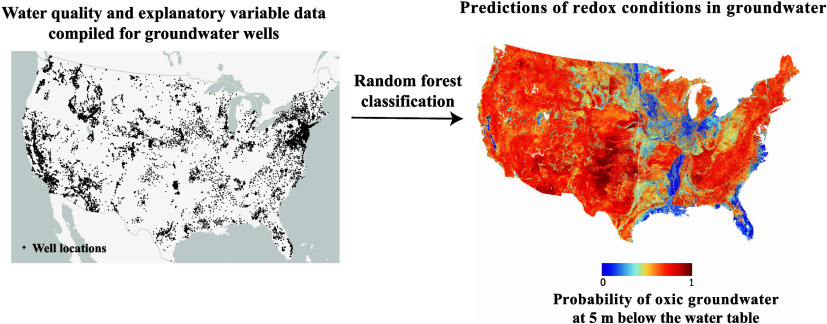

Redox conditions in groundwater may markedly affect the fate and transport of nutrients, volatile organic compounds, and trace metals, with significant implications for human health. While many local assessments of redox conditions have been made, the spatial variability of redox reaction rates makes the determination of redox conditions at regional or national scales problematic. In this study, redox conditions in groundwater were predicted for the contiguous United States using random forest classification by relating measured water quality data from over 30,000 wells to natural and anthropogenic factors. The model correctly predicted the oxic/suboxic classification for 78 and 79% of the samples in the out-of-bag and hold-out data sets, respectively. Variables describing geology, hydrology, soil properties, and hydrologic position were among the most important factors affecting the likelihood of oxic conditions in groundwater. Important model variables tended to relate to aquifer recharge, groundwater travel time, or prevalence of electron donors, which are key drivers of redox conditions in groundwater. Partial dependence plots suggested that the likelihood of oxic conditions in groundwater decreased sharply as streams were approached and gradually as the depth below the water table increased. The probability of oxic groundwater increased as base flow index values increased, likely due to the prevalence of well-drained soils and geologic materials in high base flow index areas. The likelihood of oxic conditions increased as topographic wetness index (TWI) values decreased. High topographic wetness index values occur in areas with a propensity for standing water and overland flow, conditions that limit the delivery of dissolved oxygen to groundwater by recharge; higher TWI values also tend to occur in discharge areas, which may contain groundwater with long travel times. A second model was developed to predict the probability of elevated manganese (Mn) concentrations in groundwater (i.e., ≥50 μg/L). The Mn model relied on many of the same variables as the oxic/suboxic model and may be used to identify areas where Mn-reducing conditions occur and where there is an increased risk to domestic water supplies due to high Mn concentrations. Model predictions of redox conditions in groundwater produced in this study may help identify regions of the country with elevated groundwater vulnerability and stream vulnerability to groundwater-derived contaminants.

## Abstract 2

Redox conditions in groundwater may markedly affect the fate and transport of contaminants but are not often known. This study uses random forest classification to predict redox conditions in groundwater in the contiguous United States.

## 1 Introduction

Redox processes influence groundwater contaminant transport and potential toxicity either by directly transforming contaminants to other species^1^ or by causing the precipitation or dissolution of compounds that contain or sorb contaminants in the vadose and saturated zones.^2−4^ These processes have been shown to markedly affect the transport of nutrients,^5^ volatile organic compounds,^6^ and trace metals^7,8^ and have significant implications for human health. For example, redox-sensitive constituents are among the contaminants most likely to exceed health-based screening levels for drinking water from both domestic and public supply wells.^9−12^ While redox conditions are typically established by natural factors, anthropogenic activities can affect redox conditions and alter contaminant mobilization. For example, denitrification of nitrate from agricultural sources may lead to the mobilization of some trace metals^13,14^ and irrigation induced changes in redox conditions may mobilize arsenic.^15^ Similarly, managed aquifer recharge may shift redox conditions, causing the release of arsenic and other geogenic contaminants.^16^ Redox gradients in groundwater can also have a significant effect on the fate and lag times of contaminants transported from the landscape to groundwater and streams.^17,18^

**Figure 1:**
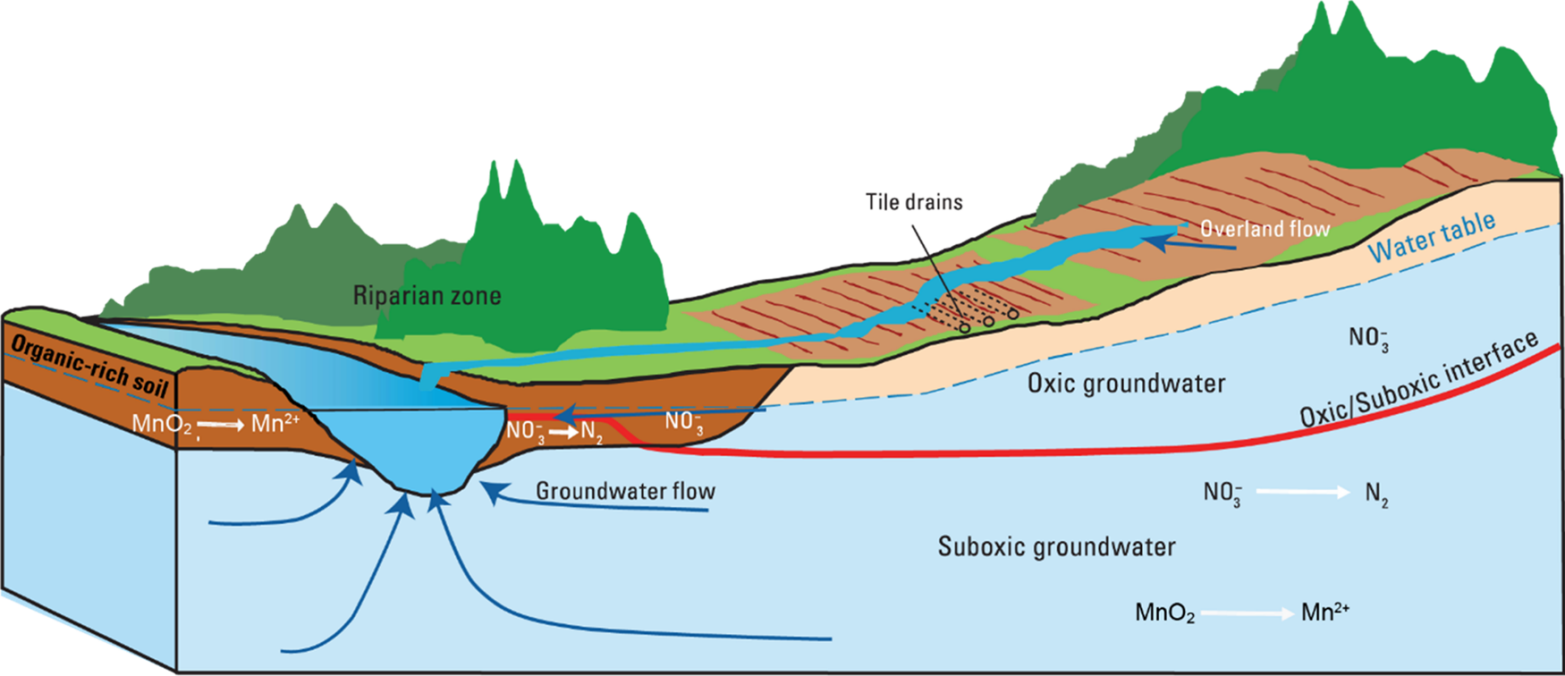
Redox conditions depend on natural and anthropogenic factors that affect the delivery of oxygen in recharging groundwater and/or the reactivity and amount of electron donors. For example, redox conditions may change as the depth below the water table increases or as organic-rich riparian zones are encountered. [Figure adapted from Wherry et al.,^28^ not subject to U.S. copyright. Published by the American Chemical Society].

The influence of redox reactions on contaminant transport underscores the importance of defining redox reaction zones in groundwater (Figure 1). Redox reactions may be abiotically or biotically mediated;^19^ however, the overall redox conditions of most aquatic environments are determined by the terminal electron accepting processes of microbial metabolism.^20^ Microbial metabolism depends on the oxidation of organic or inorganic (e.g., FeS_2_) species to generate energy for growth and maintenance, with the metabolic reaction that yields the most energy typically dominating over competing reactions.^21^ The preference for the most energetically favorable reactions results in a predictable sequence of reactions, often termed the redox ladder.^20^ Microbes will first oxidize organic carbon or reduced minerals using O_2_ as an electron acceptor through aerobic respiration. After O_2_ is depleted, facultative anaerobes will begin to use nitrate (NO_3_^–^) as an electron acceptor during denitrification. The reduction of Mn(IV), Fe(III), and sulfate will typically occur next, followed by methanogenesis. A redox classification scheme for groundwater was established using the concentrations of products and reactants in key redox reactions.^22^ A similar approach has been utilized in several aquifers across the world to assess contaminant transport, both in groundwater and streams.^23−25^ Redox conditions have also been mapped at the local scale using electrodes^26^ and at the small catchment scale (101 km^2^) using modeling.^27^

The spatial variability of redox reaction rates has made the spatially continuous determination of redox conditions at regional scales (e.g., >100,000 km^2^) problematic. As a result, until recently, studies that have estimated redox conditions on the regional scale have been rare. This has changed within the past decade as machine learning and statistical techniques have been increasingly used to predict redox conditions in aquifers around the world.^29−35^ The advancement in machine learning techniques and the availability of a national water quality data set^36^ have created an opportunity to accurately predict redox conditions in groundwater across the contiguous United States, a much larger scale than previously assessed. In this study, machine learning methods were used to relate explanatory variables representing geology, soil characteristics, hydrology, land use, and many other factors to redox-active constituent concentrations to predict redox conditions in groundwater in the contiguous United States. These predictions may be important inputs for both process-based and statistical water quality models that are designed to assess the vulnerability of groundwater and streams to contamination.^28^

## 2 Methods

Redox conditions in groundwater were predicted using measured water quality data, explanatory variables, and machine learning methods. First, redox conditions in groundwater were determined for groundwater from each well in an existing database. Redox conditions in groundwater were then related to explanatory factors by using machine learning methods. Gradient boosting machines (GBM),^37^ extreme gradient boosting (XGBOOST),^38^ and random forest^39^ were initially employed using all variables to inform our method selection process. Model performance was similar (<2% difference in percent accuracy) for all three methods. Random forest was selected for this application primarily because of the ease with which it can handle categorical variables. The water quality data set, redox classification, explanatory variables, and the steps used to apply the random forest classification (RFC) method are described below.

### 2.1 Water Quality Data and Redox Classification

Chemical data from over 43,000 wells from a previous study^36^ were examined for use as an indicator of redox conditions in groundwater in the contiguous United States (Figures SI-1 and SI-2). Sample years range from 1988 to 2017, with the most recent or most complete data set used when wells were sampled more than once. Dissolved oxygen (O_2_), nitrate or nitrite plus nitrate (NO_3_^–^), Mn, and iron (Fe) data from this data set were used to establish redox conditions in groundwater. Nitrate, Mn, and Fe samples were filtered in the field (0.45 μm, typically by using acrylic polymer or glass fiber filters).

Redox conditions have often been defined using individual concentrations, such as defining oxic conditions as occurring if dissolved oxygen concentrations exceed a certain threshold.^30^ However, a more informative classification can be attained if the redox classification is defined using multiple constituents.^40^ In this study, thresholds of Mn < 50 μg/L and Fe < 100 μg/L were deemed consistent with oxic conditions based on a previous classification system.^40^ A threshold for dissolved O_2_ of 2 mg/L, rather than 0.5 mg/L as defined previously,^40^ was used in this study to define oxic conditions based on laboratory and field data. Laboratory studies have indicated that denitrification may occur at O_2_ concentrations over 1 mg/L.^41^ Field assessments of redox conditions may be complicated by pumping and long well screens that can mix groundwater of different redox compositions.^42,43^ A compilation of field studies suggested that the threshold for denitrification of 0.5 mg/L may be too low; the relation between denitrification reaction progress and O_2_ concentrations in groundwater from over 400 wells across the United States indicated that water from nearly all wells with O_2_ concentrations below 2 mg/L showed evidence of denitrification.^5^ A threshold of 2 mg/L was also consistent with a classification tree analysis of a larger data set (872 wells) where O_2_ concentrations less than 1.93 mg/L were found to be predictive of denitrifying conditions.^44^

For the oxic/suboxic model, water from wells were classified as oxic if the following criteria were met: dissolved O_2_ ≥ 2 mg/L, Mn < 50 μg/L, and Fe < 100 μg/L. Water from wells were classified as suboxic if either nitrate-reducing (i.e., dissolved O_2_ < 2 mg/L, nitrate ≥0.5 mg/L, Mn < 50 μg/L, and Fe < 100 μg/L), Mn-reducing (i.e., dissolved O_2_ < 2 mg/L, nitrate <0.5 mg/L, Mn ≥ 50 μg/L, and Fe < 100 μg/L), or Fe-reducing (i.e., dissolved O_2_ < 2 mg/L, nitrate <0.5 mg/L, and Fe ≥ 100 μg/L) conditions were indicated. Samples with mixed redox classifications were dropped; this included samples with such low dissolved oxygen, nitrate, Mn, and Fe concentrations that a precise redox classification could not be made. Seventy percent of the data (13,723 wells) were randomly selected to construct a training data set that was used to build the oxic/suboxic model developed for this study. Data from the remaining 30% of the wells (5881 wells) formed the hold-out data set. The hold-out data set was not used to construct the model. As a result, comparing model predictions for the hold-out data set with measured observations allowed for an independent evaluation of model performance.

A second model was constructed to evaluate the presence or absence of Mn concentrations ≥50 μg/L; concentrations at or above this level are consistent with Mn-reducing conditions.^40^ No other constituents were used to indicate Mn-reducing conditions for this model to maximize the number of samples available. Mn concentration and explanatory data were available from a total of 36,515 wells, with 70% of these used for the training data set (25,482) and the remaining 30% (11,033) used for the hold-out data set.

### 2.2 Explanatory Variables

Over 200 explanatory variables representing geology, hydrology, land use, soil hydrology and chemistry, and nitrogen inputs were compiled for the random forest models that predicted redox conditions in groundwater (Table S1). Predictors were attributed to each well either by point extraction or by calculating the mean or median value of the attribute within a 500 m radius around each well. While the optimum buffer size to assess the impact of watershed characteristics on water quality may vary,^45^ the 500 m buffer size has been shown to adequately represent watershed characteristics in groundwater assessments using similar data sets.^46,47^ Variables were considered based on their potential to describe the sources of electron donors or acceptors in the vadose (e.g., soil organic carbon content, soil drainage) and saturated zones (e.g., surficial geology and subsurface lithology), reaction rates (e.g., temperature), or travel time (e.g., depth below the water table and lateral position (LP) in the watershed). Many variables are also indicators of hydrologic conditions that could identify conducive conditions for the onset of suboxic conditions such as depth to water, soil hydrology, recharge, and topographic wetness index (TWI). Several redox-sensitive constituent concentrations in soils (e.g., Fe and Mn) were also considered as indicators of soil redox environments.

Land use practices were also included as variables, as these perturbations can alter natural redox conditions in a groundwater system. For example, increased irrigation with water containing high levels of dissolved oxygen and nitrate may lead to a more oxic condition in groundwater than would otherwise occur, potentially mobilizing contaminants.^48,49^ Similarly, an increase in the amount of nitrate applied to the land surface can lead to a more oxidized environment than would occur naturally.^50^ A complete list of the variables considered for model inclusion is provided in Table S1.

### 2.3 Random Forest Classification

Random forest classification (RFC) has proven to be an effective method for predicting the probability of a classification event for a binary dependent variable in environmental applications.^33,51,52^ RFC analysis applies the random forest algorithm to classification tree analysis, resulting in many classification trees and more accurate classifications.^39,51^ The random forest algorithm combines “bagging” with random selection of variables for each partition of a classification tree. The number of variables to consider at each partition, termed *mtry*, and the number of trees, were varied until model performance was optimized. In bagging, decision trees are created from randomly selected subsets of the training samples, with each tree yielding a prediction. These predicted values are averaged to yield the best prediction. Observations that are in the training data set but are not part of the randomly selected subset of samples used to train the model are called out-of-bag (OOB) samples and are used to test the performance of the model. Models were also evaluated using the hold-out data set.

Models were initially constructed by using all variables. However, removing variables from machine learning models has been shown to improve interpretability of the model with little loss in predictive performance.^28,33,53^ The relative importance of each variable was determined to aid in the selection of variables and interpretation of the final model. Variable importance was determined using the mean decrease in accuracy (MDA) observed between model results and results determined by randomly permuting a selected variable.^39^ MDA is calculated by determining the prediction error rate for classification on the out-of-bag portion of the data in the full model and after permutation of an individual predictor variable. The differences between these error rates are then averaged over all trees and normalized by the standard deviation of the differences to calculate the MDA. These steps are repeated for each remaining variable. Higher MDA values suggest that a variable has greater importance in comparison to variables with lower MDA values. To minimize the computer processing time, only the top 25 variables based on MDA were evaluated for consideration in the final model. If any of these 25 variables were highly correlated (*r* ≥ 0.7), the lower ranked correlated variable was removed from consideration for model inclusion. After removing correlated variables, the top 20 variables were evaluated for model inclusion using recursive feature elimination (RFE) in the R *caret* package;^54^ the USGS Tallgrass supercomputer was used to run these simulations.^55^ RFE uses backward feature elimination within a 10-fold cross validation (CV) routine performed on the training data to reduce the number of variables. During RFE, the least important variable is removed, a new model is constructed, and variable importance is reranked. This process was continued until a 10-variable model was generated. Models were then tuned using the *caret* package^54^ to determine the optimum values for *mtry* and the number of trees. The final parameters for the oxic/suboxic model were *mtry* = 5 and the number of trees = 4000. For the Mn model, the final parameters were *mtry* = 6 and the number of trees = 3000. The final 10-variable models were used to predict redox conditions in groundwater at 1 km resolution across the contiguous United States (CONUS). All input variables, model output, and the R scripts used to construct the models are provided in the data release associated with this article.^56^

Partial dependence plots were constructed to illustrate the marginal effect a variable has on the probability of an event (e.g., oxic conditions occur). Partial dependence plots for a target variable were constructed by holding all other variables at their average value and using the random forest classification model to calculate the marginal effect that the target variable has on class probabilities. Marginal effects have proven to be useful in describing the average effect of changes in explanatory variables on the change in the probability of outcomes in nonlinear models.^51,57^

Model performance was evaluated in the OOB and hold-out data sets by determining the percentage of samples that were correctly classified and using Cohen’s κ statistic. For both measures, it is necessary to classify each prediction as either oxic or suboxic. Samples were classified as oxic if the predicted probability of oxic water was ≥50% and suboxic if the predicted probability of oxic water was <50%. Predictions were then compared to observed conditions in both the OOB and hold-out data sets to determine the percent of observations that were correctly classified. Model sensitivity was determined by calculating the percentage of oxic observations that were correctly classified as oxic. Model specificity was determined by calculating the percentage of suboxic observations that the model correctly classified as suboxic. Performance measures were similarly calculated for the Mn model.

Cohen’s κ statistic, κ, is a statistical measure of agreement that corrects for agreement due to chance and is an effective measure for comparing presence/absence models.^58^ A κ value of <0.2 indicates that there is poor or only slight agreement between predictions and observations other than what would be expected by chance, values between 0.2 and 0.4 indicate fair agreement, values between 0.4 and 0.6 indicate moderate agreement, and values above 0.6 indicate substantial or near perfect agreement.^59^

## 3 Results and Discussion

### 3.1 Oxic/Suboxic Model

The 10 variables included in the oxic/suboxic model describe four general characteristics: hydrology, geology, soil characteristics, and hydrologic position (Table 1). Surficial geology and subsurface lithology were both included in the model, likely due to two factors: (1) the influence of geology on hydraulic conductivity and its effect on recharge and (2) the amount of electron donors present in the geologic deposits. For example, partial dependence plots suggest that volcanic deposits, carbonate residual materials, and glaciofluvial deposits were the most favorable environments for oxic conditions, likely due to the relatively rapid infiltration and low concentration of electron donors in these environments. Conversely, silty and clayey glacial till, alluvial deposits, and proglacial deposits were among the least likely to have oxic conditions. These deposits are relatively young and either limit recharge, like glacial till, or are associated with settings (e.g., stream deposition) that are associated with long groundwater residence times and greater reactivity and abundance of electron donors. These factors may also affect the downward migration of a natural weathering front.^25^ The lithology variable described deeper sediments than did the surficial geology variable. Unconsolidated sand and gravel aquifers, igneous and metamorphic aquifers, and volcanic aquifers were related to an increased likelihood of oxic conditions. Conversely, sedimentary aquifers were associated with suboxic conditions, suggesting an increased source of electron donors in this environment.

**Table 1:** Variables Included in the Oxic/Suboxic Model^a^

variable	mean decrease in accuracy	source
Geology		
surficial geology	359	Soller et al.^60^
subsurface lithology	295	Kauffman et al.^61^
Soil Hydrology		
vadose zone water content	358	Zell and Sanford^62^
well-drained soils	330	Wieczorek^63^
soil grain size (#10 sieve)	240	Wieczorek^63^
poorly drained soils	214	Wieczorek^63^
Watershed Hydrology		
base flow index (BFI)	309	Wolock^64^
topographic wetness index	196	Wolock^65^
Hydrologic Position		
lateral position within eighth-order watershed	289	Belitz, Moore, Arnold, Sharpe, and Starn^66^
depth below the water table	235	Zell and Sanford^62^

a The relative influence of each variable, based on mean decrease in accuracy (MDA), is also provided. Variables with higher MDA values have greater importance for model performance than variables with lower MDA values. More information on variables is provided in Table S1.

**Figure 2:**
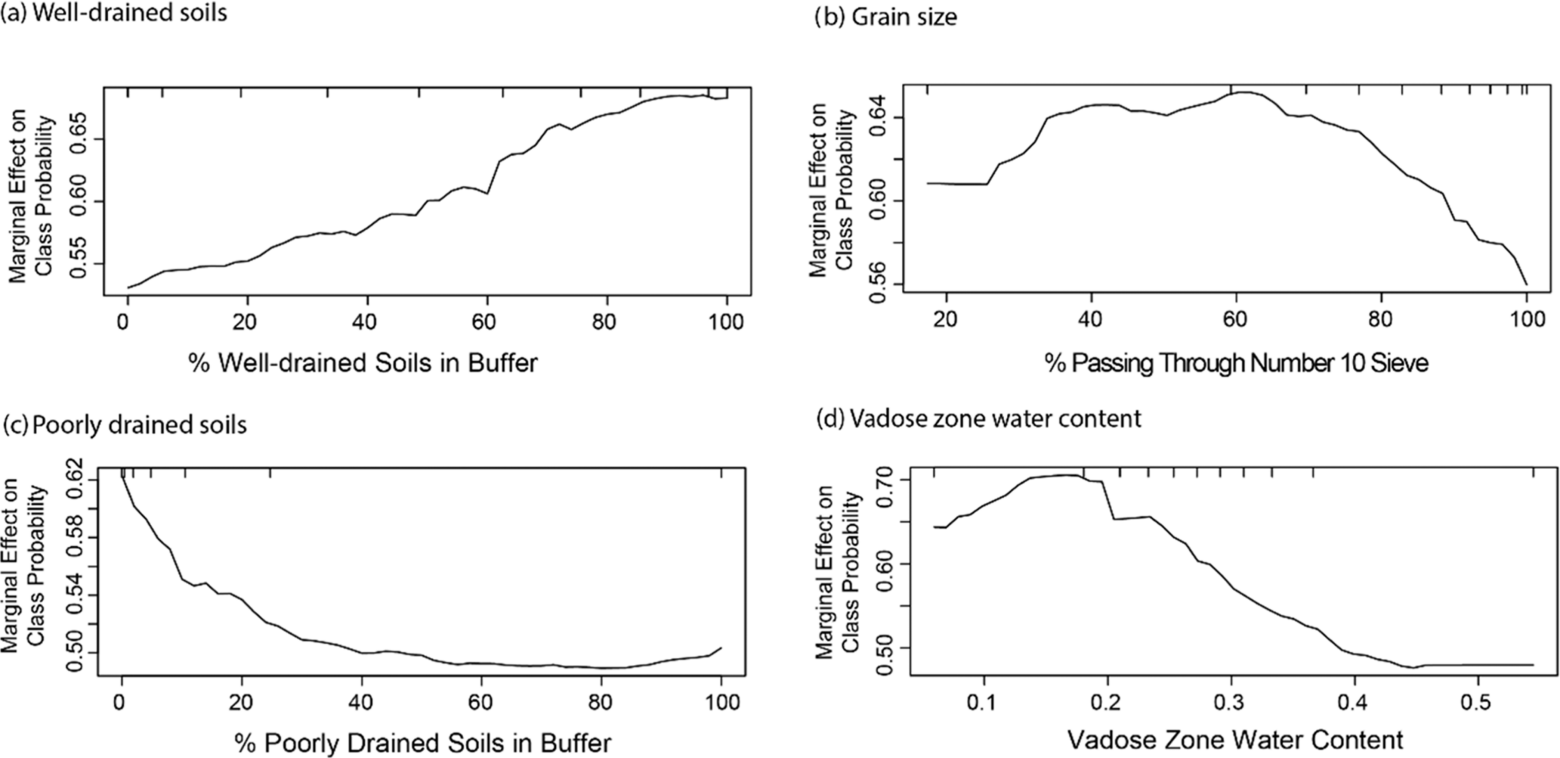
Partial dependence plots for soil variables showing the marginal effect of a single variable on the predicted probability of oxic groundwater. Soil variables in the model are (a) % of well-drained soils within buffer surrounding a well, (b) % of soil passing through a number 10 sieve, (c) % of poorly drained soils within buffer, and (d) vadose zone water content. Tick marks represent deciles of data in the training data set.

Four of the 10 variables in the oxic/suboxic model described soil properties, specifically drainage, grain size, and water content. A sharp increase in the likelihood of oxic conditions was observed as the percentage of well-drained soils increased, while a decrease in the likelihood of oxic conditions was observed as the percentage of poorly drained soils increased (Figure 2). The likelihood of oxic conditions also decreased as the percentage of fine-grained sediment increased (Figure 2). Fine-grained sediments often have lower hydraulic conductivity than coarser sediments, so differences in recharge may have been responsible for these relations. The likelihood of oxic conditions also generally decreased as vadose zone water content increased. The relations between oxic conditions and these four variables were likely driven by the interplay between recharge and redox transformations. Recharge provides aquifers with dissolved oxygen, which may be consumed by microbial processes. If recharge rates are high, oxic conditions are more likely.

**Figure 3:**
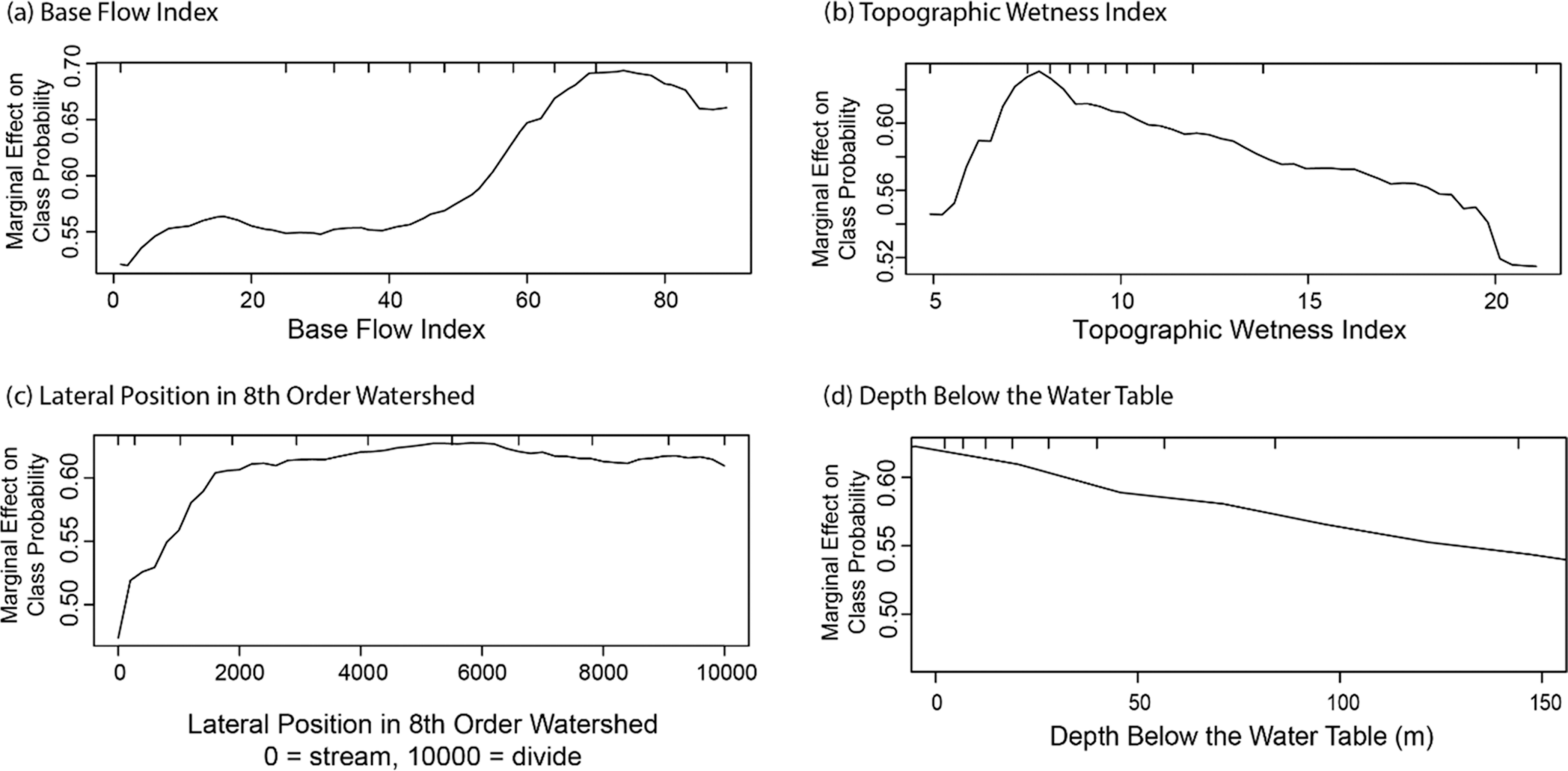
Partial dependence plots for hydrology and hydrologic position variables showing the marginal effect of a single variable on the predicted probability of oxic groundwater. Hydrology and hydrologic position variables in the model are (a) base flow index, (b) topographic wetness index, (c) lateral position in eighth-order watershed, and (d) depth below the water table. Tick marks represent deciles of data in the training data set. Some outlier values are not shown.

Four variables describing hydrology and/or hydrologic position were included in the oxic/suboxic model. The base flow index (BFI) is the percentage of annual streamflow that is derived from base flow. Oxic conditions were more likely as BFI increased, particularly when BFI values exceeded 60% (Figure 3). This finding is consistent with streambed measurements of dissolved oxygen in a previous study, where streambed samples in streams with low to moderate BFI values (<60%) had median dissolved oxygen concentrations near or below 2 mg/L.^67^ In contrast, the dissolved oxygen concentrations in streams with high BFI values typically had median streambed dissolved oxygen concentrations above 6 mg/L.^67^ High BFI watersheds are less likely to have confining layers in the shallow subsurface and more likely to have a stronger connection between aquifers and streams than low BFI watersheds. As a result, in areas with similar topography and climate, high BFI watersheds are expected to have higher recharge than low BFI watersheds.

The second hydrology variable in the oxic/suboxic model was the topographic wetness index (TWI). TWI is a measure of the topographic control of hydrological processes.^68^ Areas with high values of TWI are typically more susceptible to saturated land surfaces and are more likely to produce overland flow. TWI has been used to predict soil moisture content,^69^ assess flood risk,^70,71^ predict mean transit times of surface water and groundwater,^72,73^ and explain ecosystem conditions.^74^ In this model, higher values of TWI typically resulted in a lower likelihood of oxic conditions (Figure 3). High TWI values are more likely to be in near-stream environments that may be rich in electron donors^75^ and have longer transit times than areas with low TWI values. Both of these factors favor reducing conditions. The TWI metric differs from BFI in that TWI relies on the topography of a watershed, while BFI values are based on measured streamflow during varying flow regimes.

Hydrologic position is expected to have a significant influence on redox conditions, since longer travel times allow for more time for redox reactions to occur. In fact, changes in redox-sensitive constituents as a function of age or depth have been used to estimate redox reaction rates in groundwater systems.^5,25,76^ Depth below the water table was included in the oxic/suboxic model with the likelihood of oxic conditions decreasing as depth below the water table increased (Figure 3). For areas with similar amounts of recharge, groundwater age is expected to increase as depths below the water table increase.^73,77^ While depth below the water table provides a key variable to explain the hydrologic position of a groundwater sample, it does not consider the relative position of a sample location within a watershed. Due to the convergence of flow paths in a discharge area, water from wells with similar depths below the water table will tend to be much older near the stream than near the watershed divide. A relatively new nationally available data set, the lateral position in a watershed, addresses this issue.^66^ Lateral position (LP) is defined as the relative position of a point in a watershed. LP is calculated for each stream order by dividing the shortest horizontal distance to the stream by the shortest horizontal distance from the stream to the divide, with this value then multiplied by 10,000.^78^ As a result, LP is dimensionless, with values varying from 0 at the stream to 10,000 at the divide. While LP values from first-order (LP1) to ninth-order (LP9) streams were considered, lateral position for eighth-order streams (LP8) was the only LP variable that was one of the top 10 predictors for the oxic/suboxic model and therefore the only LP variable included in the final model. The likelihood of oxic conditions was lowest closest to the stream, with oxic conditions increasing quickly to about a fifth of the way up the watershed (LP = 2000) and then increasing more slowly (Figure 3). A lower likelihood of oxic conditions when LP8 values were low is consistent with previous work showing the increased likelihood of suboxic conditions as streams are approached^79,80^ due to an increase in electron donors in riparian zones.^81^ However, it should be noted that these studies often documented suboxic conditions near lower-order streams than eighth-order. We speculate that LP8 was selected for model inclusion because suboxic zones adjacent to eighth-order streams were typically wider than lower-order streams and/or because the effects of lower-order streams were captured by other variables. An increase in groundwater travel times may also contribute to the relation between LP8 and redox conditions since it may be expected that groundwater ages may increase as discharge areas are approached. Interestingly, LP8 was a weak predictor of groundwater ages in a recent study in the Great Lakes, with LP values for lower-order streams better at predicting age.^82^

### 3.2 Manganese Model

The variables included in the Mn model describe the same four general characteristics as those in the oxic/suboxic model: hydrology, geology, soil characteristics, and hydrologic position (Table 2). Similar to the oxic/suboxic model, surficial geology and subsurface lithology were both included in the Mn model, likely due to their influence on hydraulic conductivity and electron donor abundance. For example, partial dependence plots suggest that sedimentary deposits were one of the most favorable lithologies for Mn-reducing conditions, likely due to a higher concentration of electron donors in this environment. For the surficial deposits, partial dependence plots suggest that fine-grained sediments and biological sediments (e.g., calcareous materials) were more likely to have Mn-reducing conditions.

**Table 2:** Variables Included in the Mn Model^a^

variable	mean decrease in accuracy	source
Geology		
surficial geology	398	Soller, Reheis, Garrity, and Van Sistine^60^
subsurface lithology	225	Kauffman, Degnan, Belitz, Stackelberg, and Erickson^61^
Soil Hydrology		
somewhat poorly drained soils	171	Wieczorek^63^
Watershed hydrology		
base flow index	412	Wolock^64^
air temperature	329	PRISM Climate Group^83^
depth to water	337	Zell and Sanford^62^
topographic wetness index	229	Wolock^65^
Hydrologic Position		
depth below the water table	478	Zell and Sanford^62^
distance to drainage eighth-order watershed	311	Belitz, Moore, Arnold, Sharpe, and Starn^66^
lateral position within eighth-order watershed	304	Belitz, Moore, Arnold, Sharpe, and Starn^66^

a The relative influence of each variable, based on mean decrease in accuracy (MDA), is also provided. Variables with higher MDA values have greater importance for model performance than variables with lower MDA values.More information on variables is provided in Table S1.

**Figure 4:**
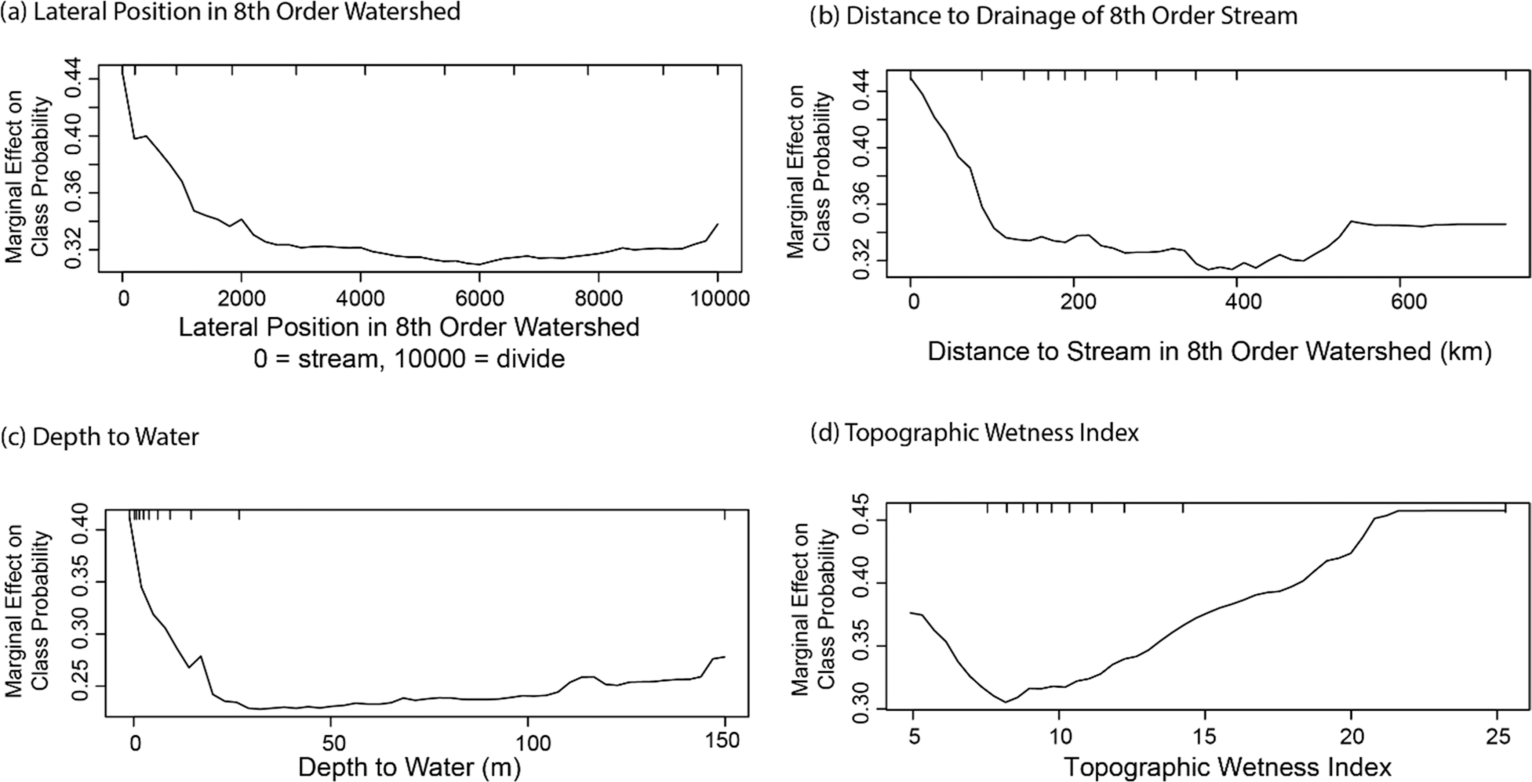
Partial dependence plots showing the marginal effect of a single variable on the predicted probability of a Mn concentration in groundwater ≥50 μg/L. All of these plots illustrate that close proximity to shallow water table environments is related to an increased probability of elevated Mn concentrations. Tick marks represent deciles of data in the training data set.

**Figure 5:**
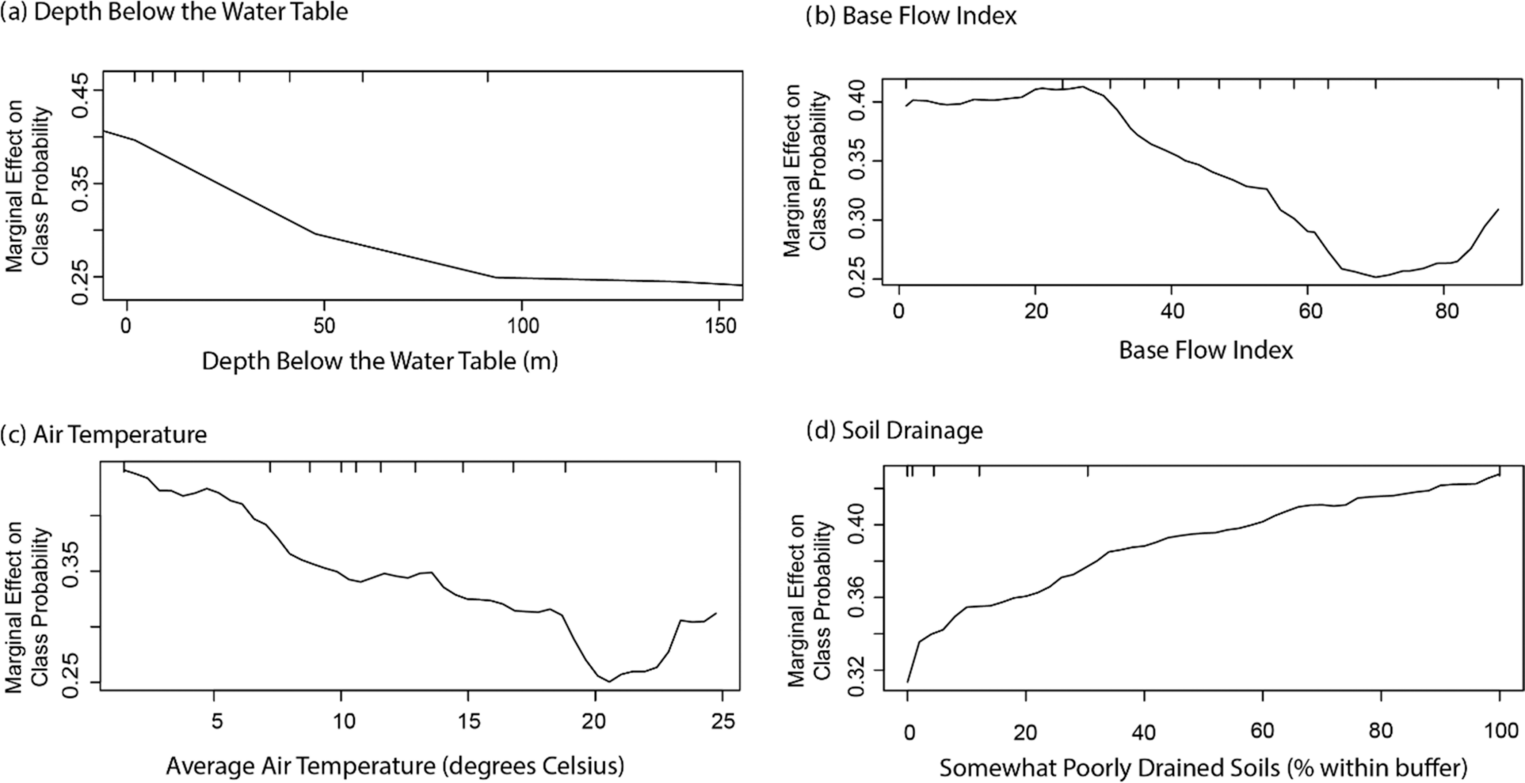
Partial dependence plots showing the marginal effect of a single variable on the predicted probability of a Mn concentration in groundwater ≥50 μg/L. These plots illustrate the effect of hydrologic position, watershed drainage properties, and temperature on the predicted probability of elevated Mn concentrations. Tick marks represent deciles of data in the training data set. Some outlier values are not shown.

Several variables in the Mn model were factors that may be related to transport or reactions occurring between the land surface and the water table. Proximity to streams is an important predictor of elevated Mn, with two variables representing this feature in the model: lateral position in an eighth-order watershed and distance to an eighth-order watershed. Partial dependence plots for both of these variables suggest that the probability of elevated Mn concentrations increases as streams are approached (Figure 4). In fact, 26% of wells with high Mn concentrations (>300 μg/L) in this data set were found within 500 m of a river.^36^ Manganese reduction coupled with the oxidation of organic carbon has been well documented,^84^ with previous studies finding high Mn concentrations in near-stream environments, likely due to the presence of organic-rich sediments in alluvial aquifers.^85,86^ Higher probabilities of elevated Mn in groundwater at shallow depth to water (Figure 4) and depth below the water table (Figure 5) are also consistent with the transport of organic carbon or other processes that may occur in the near water table environment, such as the reductive dissolution of Mn-oxides that accumulate in sediments near the water table.^87^

Three of the remaining variables are related to drainage conditions within 500 m of a well (Figures 4 and 5). Partial dependence plots show that the probability of elevated Mn generally increases as topographic wetness index values and the percentage of somewhat poorly drained soils increase, which may be expected since poor drainage conditions often lead to suboxic conditions and the reductive dissolution of Mn-oxides. The relation between elevated Mn and BFI may also be related to watershed drainage conditions. The decrease in the probability of elevated Mn as BFI increases may be because high BFI watersheds are more likely to have oxic water due to the well-drained conditions.^67^ The tendency for the probability of elevated Mn to decrease as air temperature increases (Figure 5) is not expected since O_2_ depletion in groundwater and the onset of Mn-reducing conditions have been shown to occur more quickly at higher temperatures.^88−90^ While a definitive explanation of this relation is not possible, less vegetation and organic carbon in hot, arid parts of the country may contribute to the tendency for lower Mn concentrations in these environments.

No variables representing nitrogen loading or land use were included in the oxic-suboxic or Mn models. This suggests that natural factors, not anthropogenic factors, are the dominant influences on redox conditions, at least at the scale at which this study was done. There are many notable examples of anthropogenic influences on redox conditions in the literature from both point^24,91,92^ and nonpoint sources.^13,16^ Some of these anthropogenic factors likely affected redox conditions in some samples; however, the effects were not sufficient to be selected as a major driver of redox conditions in this large data set.

### 3.3 Performance and Limitations

The oxic/suboxic model was tested by comparing predicted probabilities in the out-of-bag data (OOB) and hold-out data sets with observed classifications. The oxic/suboxic model correctly predicted the redox classification in 78 and 79% of samples in the OOB and hold-out data sets, respectively. κ values of 0.52 and 0.54 for the OOB and hold-out data sets, respectively, indicate moderate agreement. Sensitivity and specificity rates in the OOB data set were 86 and 65%, respectively. Similar sensitivity and specificity rates were observed in the hold-out data set (i.e., 88 and 65%). The high sensitivity values suggest that model predictions of oxic conditions have a high degree of accuracy (i.e., low false-positive rate). Conversely, predictions of suboxic conditions were more likely to be incorrect (i.e., moderate false-negative rate) than were predictions of oxic conditions.

The Mn model correctly classified whether samples were ≥50 μg/L or <50 μg/L in 78 and 80% of the samples in the OOB and hold-out data sets, respectively. κ values of 0.48 and 0.49 for the OOB and hold-out data sets, respectively, indicate moderate agreement. Sensitivity and specificity rates in the OOB data set were 56 and 89%, respectively. Similar sensitivity and specificity rates were observed in the hold-out data set (i.e., 58 and 89%). The high specificity values indicate that model predictions of Mn concentrations in groundwater <50 μg/L have a high degree of accuracy (i.e., low false-negative rate). Lower sensitivity rates for the Mn model than in the oxic/suboxic model were expected because events (i.e., Mn ≥ 50 μg/L for the Mn model and oxic classification for the oxic/suboxic model) were a smaller fraction of the sample population in the Mn data set than in oxic/suboxic data set.

There are several key limitations to the models developed to predict redox conditions in the CONUS. First, models were created by using available data that were not evenly distributed across the nation (Figures SI-1 and SI-2). Predictions may be less accurate where data are sparse and in all cases, model predictions should not take precedence over local knowledge of redox conditions in groundwater. The primary utility of model predictions is to provide guidance in areas where local assessments of redox studies do not exist. Second, redox conditions can change markedly as depth below the water table increases but the rate of change varies spatially.^18,76^ Depth below the water table is based on well depth and previously modeled estimates of the depth to water. Well depth was used instead of depth to the midpoint of the screened interval because screen length was not available for many wells in our data set. Relying on well depth instead of the depth to the midpoint of a screened interval may result in model predictions that overestimate the depth of a redox condition, particularly if screen lengths are large. Inaccuracies in modeled estimates of depth to water may also affect model predictions developed in this paper. Third, temporal changes in redox-sensitive concentrations were not examined, but previous studies suggest that temporal changes are likely to be small in most cases.^18,93^ Further, data^94^ from a recently completed groundwater trends study^95^ suggest that changes in redox classification over a decadal time scale are not common. Fourth, models may be improved if the buffer radii of variables describing surface properties are optimized and if more information is available on conditions at depth. Most variables in this study describe surface or near-surface properties. The presence of confining layers or layers rich in electron donors at depth will affect redox conditions but could not be characterized with currently available data on the CONUS scale. Some examples of data that could improve predictions include CONUS-scale estimates of apparent groundwater age, depth to a confining layer, and the location of formations that are rich in electron donors. Last, while redox-sensitive concentration thresholds used in this study have been widely applied,^40,96^ these thresholds vary as a function of pH, a feature that is not included in this classification system. As a result, predictions of redox conditions may be less accurate under extreme pH conditions (e.g., acid mine drainage sites).

### 3.4 Spatial Distribution of Redox Conditions and Implications for Vulnerability Assessments

**Figure 6:**
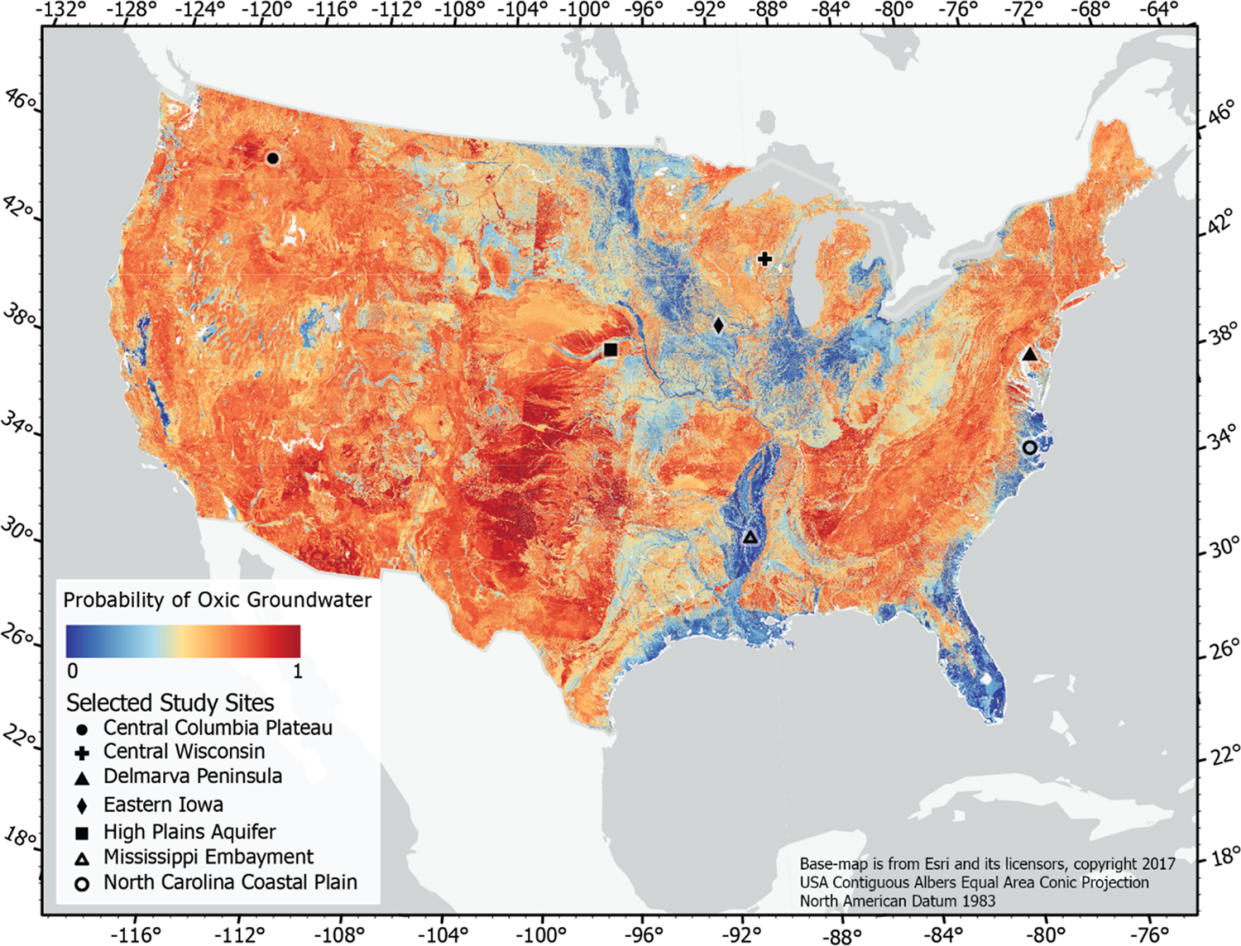
Map depicting the predicted probability of oxic conditions in shallow groundwater. Predictions were made by assuming depth below the water table is 5 m. Selected study sites are examples of areas where detailed assessments of redox conditions were conducted using water quality data. The relations between predictions and observations at these sites are discussed in the text. A map of the predicted probability of oxic water in deeper groundwater is provided in the Supporting Information.

**Figure 7:**
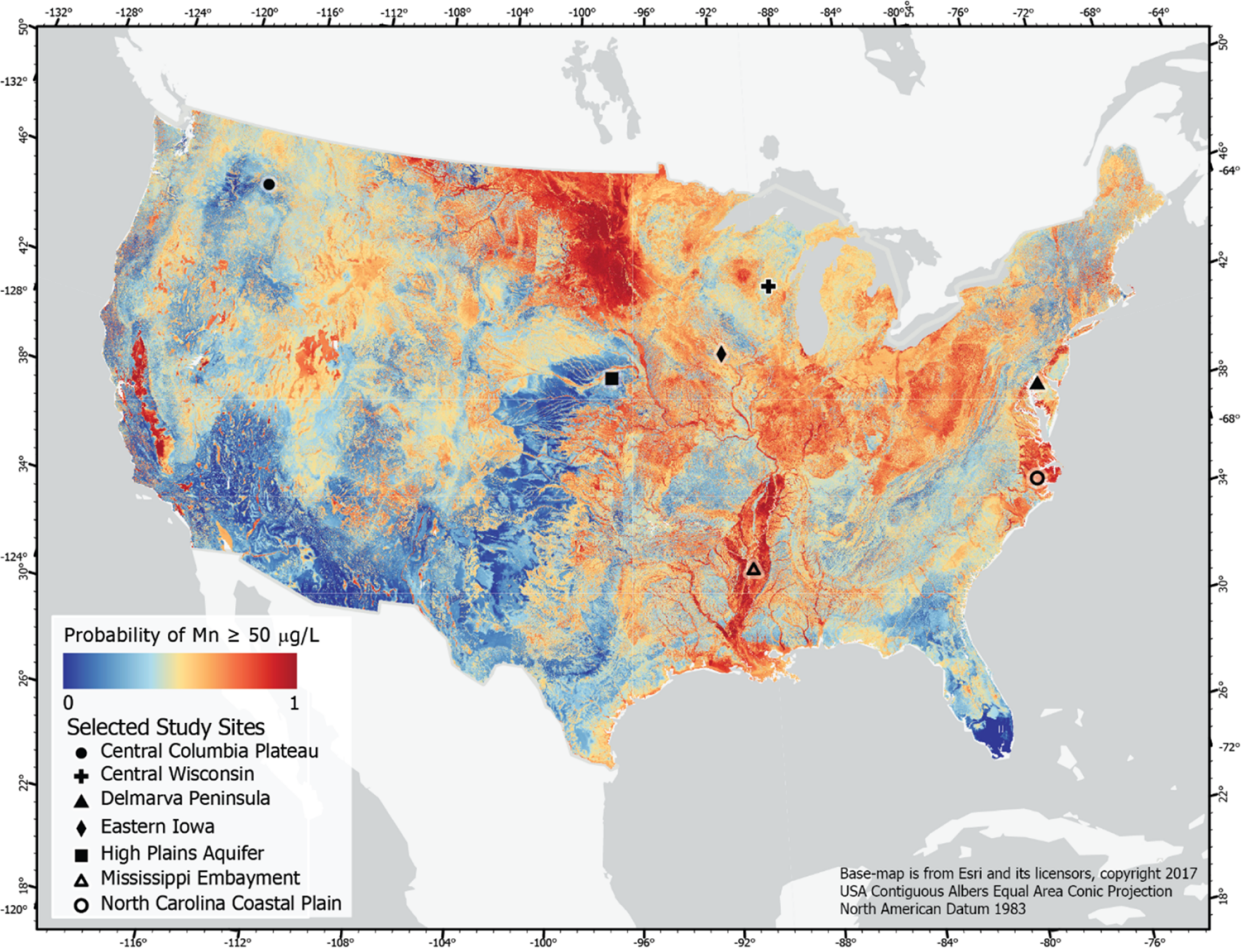
Map depicting the predicted probability of a manganese concentration ≥50 μg/L in shallow groundwater. Predictions were made by assuming depth below the water table is 5 m. Selected study sites are examples of areas where detailed assessments of redox conditions were conducted using water quality data. The relations between predictions and observations at these sites are discussed in the text. A map of the predicted probability of a manganese concentration ≥50 μg/L in deeper groundwater is provided in the Supporting Information.

The spatial distribution of oxic/suboxic conditions provides vital information for assessing the susceptibility of areas to redox-sensitive contaminants (Figure 6). The Mn model is not discussed in detail for brevity and because much of the discussion of the oxic–suboxic model also applies to the Mn model. Changes in redox conditions with depth were assessed by examining partial dependence plots (Figures 3d and 5a) and by comparing maps of predicted redox conditions at different depths below the water table. Partial dependence plots suggest that the probability of oxic conditions (Figure 3d) and elevated Mn decreases with depth (Figure 5a). When examined spatially, changes in redox conditions with depth were not dramatic, with areas with a high probability of oxic water generally decreasing as the depth below the water table increased (e.g., Figures 6 and SI-3). Similarly, areas with a high probability of a Mn concentration above 50 μg/L also decreased as the depth below the water table increased (Figures 7 and SI-4). Redox predictions were compared to selected field-based redox characterizations that were conducted as part of a national water quality program.^95,97^ Most of these studies followed contaminants along a transect from recharge in upland areas to discharge in streams. Comparing these field-based redox characterizations to model predictions provides insight into the performance of the models and potential applications of model results.

Redox conditions in shallow groundwater within glacial deposits in the Midwestern United States were highly variable. Suboxic areas are more likely in portions of the Midwestern United States, including large portions of Indiana, Illinois, and Iowa. Many of these areas have poorly drained soils and glacial till, with many agricultural areas using tile drains to allow for crop production. Transect studies conducted in areas with a low probability of oxic conditions in Indiana,^98^ Iowa,^18^ and Minnesota^25,99^ confirm the presence of suboxic conditions in shallow groundwater in these regions (see Eastern Iowa in Figure 6). Even with high nitrogen loading in these areas, suboxic conditions often lead to low nitrate concentrations in groundwater,^18^ but may provide conducive conditions for the mobilization of phosphorus in groundwater.^100^ Conversely, portions of the upper Midwest (see Central Wisconsin in Figure 6) and on the Delmarva Peninsula (see Figure 6) have a higher probability of oxic conditions due to more well-drained sediments; this finding is supported by the deep penetration of oxic conditions in these areas.^101−103^ These areas are much more likely to provide legacy nitrate to streams.^67^ Exceptions to these predictions are expected when localized confining areas are present.^104^

Suboxic conditions were predicted for the North Carolina coastal plain, an area that often has poor drainage and an ample supply of organic carbon. Predicted probabilities of oxic conditions were lower in the outer coastal plain than in the inner coastal plain. This is consistent with a previous assessment of redox conditions that suggested that suboxic conditions are prevalent in this area, particularly in the outer coastal plain where dissolved organic carbon contents were high.^105^ Prevalent suboxic conditions on the coastal plain of North Carolina likely contributes to the low risk of nitrate contamination of private wells in this area.^106^

The model correctly identified the Central Columbia Plateau and the High Plains aquifer as areas that are likely to be oxic. Both of these areas have extensive nitrate contamination in shallow groundwater and little denitrification due to the prevalence of oxic conditions.^107^ Conversely, discharge areas are more likely to be suboxic and the model correctly predicts that suboxic conditions are likely in the Mississippi Embayment^108,109^ and in the trough of the Central Valley of California.^110^ Similarly, the high probability of Mn concentrations ≥50 μg/L in the Mississippi Embayment (Figure 7) is consistent with previous work.^111^

Predicted probabilities of oxic conditions should prove useful for assessing the vulnerability of groundwater to redox-sensitive contaminants. Redox-sensitive contaminants, such as nitrate and arsenic, are among the most common constituents that exceed maximum contaminant levels (MCLs) in public drinking water supplies.^11^ Sampling of domestic wells for water quality constituents in the United States generally occurs less frequently than public water supplies because domestic wells are not regulated at the federal level. A nationwide sampling of 2167 domestic wells in the United States suggested that two redox-sensitive contaminants, arsenic and Mn, were among the most likely to exceed maximum contaminant levels (MCLs) or secondary MCLs.^10^ In California, people served by water from domestic wells may face greater water quality concerns than those served by public water supplies, with nitrate, arsenic, and chromium(VI) as the major contaminants.^112^ Given the prevalence of elevated concentrations of redox-sensitive contaminants, predictions of redox conditions provided in this study should be useful in prioritizing monitoring efforts, particularly for domestic wells, since they may pose a greater risk to human health and are often not regularly monitored. Predictions of redox conditions in groundwater may also prove useful as an explanatory variable in statistical or machine learning models that predict concentrations for a specific constituent (e.g., nitrate) and may also help estimate reaction rates for process-based models.

Predictions of redox conditions in groundwater may also inform estimates of redox-sensitive concentrations in stream base flow; lack of information on reaction rates is considered a significant data gap for predicting nitrate inputs to streams from groundwater.^113^ Model results presented here can help predict whether denitrification will occur prior to discharge. For example, streams that are in watersheds that are dominated by oxic water may be more likely to have high nitrate concentrations during base flow conditions.^28^ These oxic watersheds may also be more likely to discharge nitrate to streams that recharged the aquifer a decade or more ago (i.e., legacy nitrate). As a result, redox predictions may help with spatial targeting of conservation measures, a key strategy for improving water quality when legacy issues are present.^114^

## Supporting Information Available

Table that lists all of the variables considered in the models constructed in this paper; maps showing sample locations; and maps showing predictions of redox conditions at depth (PDF)

## Notes

Model input and output files, R scripts, and other information on model construction are provided in a data release associated with this publication.^56^
