# Supplementary material for: Predicting Redox Conditions in Groundwater at a National Scale Using Random Forest Classification
Source: Environ Sci Technol. 2024 Mar 7;58(11):5079–92. doi: 10.1021/acs.est.3c07576 (PMC10956438; doi:10.1021/acs.est.3c07576)
Supplement: Supplementary file 1 — es3c07576_si_001.pdf [file es3c07576_si_001.pdf]

Supporting Information for:

Predicting Redox Conditions in Groundwater at a National Scale Using Random Forest Classification

Anthony J. Tesoriero,* U.S. Geological Survey, 601 SW 2nd Avenue, Suite 1950

Portland, OR 97204, tesorier@usgs.gov

Susan A. Wherry, U.S. Geological Survey, 601 SW 2nd Avenue, Suite 1950, Portland, OR 97204

Danielle I. Dupuy, U.S. Geological Survey, 6000 J Street, Placer Hall, Sacramento, CA 95819

Tyler D. Johnson, U.S. Geological Survey, 4165 Spruance Road, Suite 200, San Diego, CA 92101

Supporting information includes 1 table and 4 figures.

Note: Model input and output files, R scripts, and other information on the construction of random forest models that predict redox conditions in the contiguous United States are provided in a data release associated with this publication.[1]

*Corresponding author

Disclaimer: Any use of trade, firm, or product names is for descriptive purposes only and does not imply endorsement by the U.S. Government.

Table S1. List of variables that were tested as predictors of redox conditions in groundwater using random forest classification (RFC). The ten variables selected for both the oxic/suboxic and the manganese RFC models are described in the text of the manuscript.

Variable Name	Description	Units	Attribution Type	Reference
**Well Characteristics**				
DBW	Depth below the water table	m	Well	DBW = Well depth – depth to water. McMahon et al., 2019[2] for well depth and Zell and Sanford (2020)[3] for depth to water.
**Hydrology**				
LP1	Relative position of a point between the stream and its watershed divide (first order streams and larger X 10,000)	Unitless	Point	Belitz et al., 2019[4]
LP2	Relative position of a point between the stream and its watershed divide (second order streams and larger X 10,000)	Unitless	Point	Belitz et al., 2019[4]
LP3	Relative position of a point between the stream and its watershed divide (third order streams and larger X 10,000)	Unitless	Point	Belitz et al., 2019[4]
LP4	Relative position of a point between the	Unitless	Point	Belitz et al., 2019[4]

	stream and its watershed divide (fourth order streams and larger X 10,000)			
LP5	Relative position of a point between the stream and its watershed divide (fifth order streams and larger X 10,000)	Unitless	Point	Belitz et al., 2019[4]
LP6	Relative position of a point between the stream and its watershed divide (sixth order streams and larger X 10,000)	Unitless	Point	Belitz et al., 2019[4]
LP7	Relative position of a point between the stream and its watershed divide (seventh order streams and larger X 10,000)	Unitless	Point	Belitz et al., 2019[4]
LP8	Relative position of a point between the stream and its watershed divide (eighth order streams and larger X 10,000)	Unitless	Point	Belitz et al., 2019[4]
LP9	Relative position of a point between the stream and its watershed divide (ninth order streams and larger X 10,000)	Unitless	Point	Belitz et al., 2019[4]
DSD1	Distance from stream to watershed divide (first order streams and larger)	m	Point	Belitz et al., 2019[4]
DSD2	Distance from stream to watershed	m	Point	Belitz et al., 2019[4]

	divide (second order streams and larger)			
DSD3	Distance from stream to watershed divide (third order streams and larger)	m	Point	Belitz et al., 2019[4]
DSD4	Distance from stream to watershed divide (fourth order streams and larger)	m	Point	Belitz et al., 2019[4]
DSD5	Distance from stream to watershed divide (fifth order streams and larger)	m	Point	Belitz et al., 2019[4]
DSD6	Distance from stream to watershed divide (sixth order streams and larger)	m	Point	Belitz et al., 2019[4]
DSD7	Distance from stream to watershed divide (seventh order streams and larger)	m	Point	Belitz et al., 2019[4]
DSD8	Distance from stream to watershed divide (eighth order streams and larger)	m	Point	Belitz et al., 2019[4]
DSD9	Distance from stream to watershed divide (ninth order streams and larger)	m	Point	Belitz et al., 2019[4]
BFI48grd	Baseflow index (ratio of base flow to total flow)	%	Point	Wolock, 2003[5]
DTW	Depth to water	m	Buffer mean and median	Zell and Sanford, 2020[3]
VRT	Vadose zone residence time	years	Buffer mean and median	Zell and Sanford, 2020[3]
VWC	Vadose zone water content	Percent by volume, (vol. / vol.)	Buffer mean and median	Zell and Sanford, 2020[3]
Unconfined_250_transtrans	Estimated effective transmissivity of the	m^2/day	Buffer mean	Zell and Sanford, 2020[3]

	surficial groundwater system			
rech48grd	Natural recharge	mm/year	Point	Wolock, 2003[6]
MeanRC_eff_myr_Reitz	Mean effective recharge (2000-2013)	m/year	Point	Reitz et al., 2017[7]
runoff_Reitz	Mean annual quick-flow runoff (2000-2013)	m/year	Buffer mean	Reitz et al., 2017[7]
satof48	Saturation overland flow in total stream flow	%	Point	Wolock, 2003[8]
MeanStreamDensity	Density of streams for each raster cell	m/m^2	Buffer mean	USGS, 2014[9]
Distance_nearest_river	Distance to stream or water body	m	Point	USGS, 2014[9]
SubsurfContactTime_mean, SubsurfContactTime_median	Subsurface flow contact time	Days	Buffer mean and median	Wolock, 2003[8]
Runoff_TWI_mean, etc.	Topographic wetness index	ln(m)	Buffer mean, median, min, max	Wolock, 2003[8]
**Climate**				
us_ppt1981_mmyr	Precipitation (mean annual 30-year normal, 1981-2010)	mm/year	Point	PRISM Climate Group, 2014[10]
us_tave_1981_2010	Temperature (mean annual 30-year normal, 1981-2010)	C	Point	PRISM Climate Group, 2014[10]
PET_mean, PET_median	Potential evapotranspiration (PET, mean annual 1961-1990)	mm/year	Buffer mean and median	Wolock, 2003[11]
ET_Reitz	Evapotranspiration (AET, mean annual 2000-2013)	m/year	Buffer mean	Reitz et al., 2017[7]
**Soils**				
DrnClass_2_mean	Moderately well-drained soils	%	Buffer mean	Wieczorek, 2014[12]
DrnClass_3_mean	Drainage not applicable	%	Buffer mean	Wieczorek, 2014[12]

DrnClass_4_mean	Poorly drained soils	%	Buffer mean	Wieczorek, 2014[12]
DrnClass_5_mean	Somewhat excessively drained soils	%	Buffer mean	Wieczorek, 2014[12]
DrnClass_6_mean	Somewhat poorly drained soils	%	Buffer mean	Wieczorek, 2014[12]
DrnClass_8_mean	Very poorly drained soils	%	Buffer mean	Wieczorek, 2014[12]
DrnClass_9_mean	Well drained soils	%	Buffer mean	Wieczorek, 2014[12]
hydgrp_A_mean	Soil with high infiltration rate and low runoff. Soil hydrologic group A.	%	Buffer mean of raster cell percentage	Wieczorek, 2014[12]
hydgrp_AD_mean	High water table soil with drained group A and natural group D. Soil hydrologic group A/D.	%	Buffer mean of raster cell percentage	Wieczorek, 2014[12]
hydgrp_B_mean	Soil with moderate infiltration rate. Soil hydrologic group B.	%	Buffer mean of raster cell percentage	Wieczorek, 2014[12]
hydgrp_BD_mean	High water table soil with drained group B and natural group D. Soil hydrologic group B/D.	%	Buffer mean of raster cell percentage	Wieczorek, 2014[12]
hydgrp_C_mean	Soil with slow infiltration rate. Soil hydrologic group C.	%	Buffer mean of raster cell percentage	Wieczorek, 2014[12]
hydgrp_CD_mean	High water table soil with drained group C and natural group D. Soil hydrologic group C/D.	%	Buffer mean of raster cell percentage	Wieczorek, 2014[12]
hydgrp_D_mean	Soil with very slow infiltration rate and high runoff potential. Soil hydrologic group D.	%	Buffer mean of raster cell percentage	Wieczorek, 2014[12]

Hydcon_1_mean	Percent of soil unit with the natural condition of the soil component being "farmable under natural conditions"	%	Buffer mean of raster cell percentage	Wieczorek, 2014[12]
Hydcon_2_mean	Percent of soil unit with the natural condition of the soil component being "not applicable"	%	Buffer mean of raster cell percentage	Wieczorek, 2014[12]
Hydcon_3_mean	Percent of soil unit with the natural condition of the soil component being "neither wooded nor farmable under natural conditions"	%	Buffer mean of raster cell percentage	Wieczorek, 2014[12]
Hydcon_4_mean	Percent of soil unit with the natural condition of the soil component being "no" or no hydric condition present	%	Buffer mean of raster cell percentage	Wieczorek, 2014[12]
Hydcon_5_mean	Percent of soil unit with the natural condition of the soil component being "unranked"	%	Buffer mean of raster cell percentage	Wieczorek, 2014[12]
Hydcon_6_mean	Percent of soil unit with the natural condition of the soil component being "wooded under natural condition”	%	Buffer mean of raster cell percentage	Wieczorek, 2014[12]
Hydcon_7_mean	Percent of soil unit with the natural condition of the soil component being "yes, hydric conditions exist”	%	Buffer mean of raster cell percentage	Wieczorek, 2014[12]
avg_awc_mean	Area- and depth-weighted available water capacity	cm/cm	Buffer mean	Wieczorek, 2014[12]

avg_bd_mean	Area- and depth-weighted value for bulk density	g/cm^3	Buffer mean	Wieczorek, 2014[12]
avg_fc_mean	Volumetric content of soil water retained at a tension of 1/3 bar (33 kPa), expressed as a percentage of the whole soil	%	Buffer mean	Wieczorek, 2014[12]
avg_kfact_mean	Area- and depth-weighted for soil erodibility factor	Unitless	Buffer mean	Wieczorek, 2014[12]
avg_ksat_mean	Area- and depth-weighted saturated hydraulic conductivity	µm/s	Buffer mean	Wieczorek, 2014[12]
avg_kv_mean	Vertical saturated conductivity	µm/s	Buffer mean	Wieczorek, 2014[12]
AVG_NO10_mean	Area- and depth-weighted percentage of the soil fraction passing a number 10 sieve	%	Buffer mean	Wieczorek, 2014[12]
AVG_NO200_mean	Area- and depth-weighted percentage of the soil fraction passing a number 200 sieve	%	Buffer mean	Wieczorek, 2014[12]
AVG_NO4_mean	Area- and depth-weighted percentage of the soil fraction passing a number 4 sieve	%	Buffer mean	Wieczorek, 2014[12]
avg_om_mean	Area- and depth-weighted value of organic matter content	%	Buffer mean	Wieczorek, 2014[12]
avg_por_mean	Porosity = 100(1 - (moist bulk density/particle density))	%	Buffer mean	Wieczorek, 2014[12]
avg_clay_mean	Area- and depth-weighted average percent clay	%	Buffer mean of raster cell percentage	Wieczorek, 2014[12]

avg_sand_mean	Area- and depth-weighted average percent sand	%	Buffer mean of raster cell percentage	Wieczorek, 2014[12]
avg_silt_mean	Area- and depth-weighted average percent silt	%	Buffer mean of raster cell percentage	Wieczorek, 2014[12]
A_P	Phosphorus in A horizon of soil	mg/kg	Point	Smith et al., 2014[13]
C_P	Phosphorus in C horizon of soil	mg/kg	Point	Smith et al., 2014[13]
Top5_P	Phosphorus in top 5 centimeters of soil	mg/kg	Point	Smith et al., 2014[13]
A_As	Arsenic in A horizon of soil	mg/kg	Point	Smith et al., 2014[13]
C_As	Arsenic in C horizon of soil	mg/kg	Point	Smith et al., 2014[13]
Top5_As	Arsenic in top 5 centimeters of soil	mg/kg	Point	Smith et al., 2014[13]
A_C_Inorg	Inorganic carbon in A horizon of soil	wt. %	Point	Smith et al., 2014[13]
C_C_Inorg	Inorganic carbon in C horizon of soil	wt. %	Point	Smith et al., 2014[13]
A_C_Org	Organic carbon in A horizon of soil	wt. %	Point	Smith et al., 2014[13]
C_C_Org	Organic carbon in C horizon of soil	wt. %	Point	Smith et al., 2014[13]
A_C_Tot	Total carbon in A horizon of soil	wt. %	Point	Smith et al., 2014[13]
C_C_Tot	Total carbon in C horizon of soil	wt. %	Point	Smith et al., 2014[13]
A_Fe	Iron in A horizon of soil	wt. %	Point	Smith et al., 2014[13]
C_Fe	Iron in C horizon of soil	wt. %	Point	Smith et al., 2014[13]
Top5_Fe	Iron in top 5 centimeters of soil	wt. %	Point	Smith et al., 2014[13]
A_Mn	Manganese in A horizon of soil	mg/kg	Point	Smith et al., 2014[13]
C_Mn	Manganese in C horizon of soil	mg/kg	Point	Smith et al., 2014[13]
C_S	Sulfur in the C soil horizon	wt. %	Point	Smith et al., 2014[13]
A_S	Sulfur in the A soil horizon	wt. %	Point	Smith et al., 2014[13]

Top5_S	Sulfur in top 5 centimeters of soil	wt. %	Point	Smith et al., 2014[13]
**Land use/ N Inputs**				
*YEAR*_LU11 *1974, 1982, 1992, 2002, 2012*	Water land use	%	Buffer	Falcone, 2015[14]
*YEAR*_LU12 *1974, 1982, 1992, 2002, 2012*	Wetlands land use	%	Buffer	Falcone, 2015[14]
*YEAR*_LU21 *1974, 1982, 1992, 2002, 2102*	Developed - major transportation land use	%	Buffer	Falcone, 2015[14]
*YEAR*_LU22 *1974, 1982, 1992, 2002, 2012*	Developed - commercial/services land use	%	Buffer	Falcone, 2015[14]
*YEAR*_LU23 *1974, 1982, 1992, 2002, 2012*	Developed - industrial/military land use	%	Buffer	Falcone, 2015[14]
*YEAR*_LU24 *1974, 1982, 1992, 2002, 2012*	Developed – recreational land use	%	Buffer	Falcone, 2015[14]
*YEAR*_LU25 *1974, 1982, 1992, 2002, 2012*	Developed - residential, high density land use	%	Buffer	Falcone, 2015[14]
*YEAR*_LU26 *1974, 1982, 1992, 2002, 2012*	Developed - residential, low-medium density land use	%	Buffer	Falcone, 2015[14]
*YEAR*_LU27 *1974, 1982, 1992, 2002, 2012*	Developed – other land use	%	Buffer	Falcone, 2015[14]
*YEAR*_LU31 *1974, 1982, 1992, 2002, 2012*	Semi-developed - urban interface high land use	%	Buffer	Falcone, 2015[14]
*YEAR*_LU32 *1974, 1982, 1992, 2002, 2012*	Semi-developed - urban interface low-medium land use	%	Buffer	Falcone, 2015[14]
*YEAR*_LU33 *1974, 1982, 1992, 2002, 2012*	Semi-developed anthropogenic/other land use	%	Buffer	Falcone, 2015[14]
*YEAR*_LU41 *1974, 1982, 1992, 2002, 2012*	Production - mining/extraction land use	%	Buffer	Falcone, 2015[14]
*YEAR*_LU43 *1974, 1982, 1992, 2002, 2012*	Production – agricultural (crops) land use	%	Buffer	Falcone, 2015[14]
*YEAR*_LU44 *1974, 1982, 1992, 2002, 2012*	Production - pasture/hay land use	%	Buffer	Falcone, 2015[14]

*YEAR*_LU45 *1974, 1982, 1992, 2002, 2012*	Production - grazing potential land use	%	Buffer	Falcone, 2015[14]
*YEAR*_LU50 *1974, 1982, 1992, 2002, 2012*	Undeveloped land use	%	Buffer	Falcone, 2015[14]
*YEAR*_LU60 *1974, 1982, 1992, 2002, 2012*	Conservation land use	%	Buffer	Falcone, 2015[14]
nfarm_YEAR	Nitrogen from farm fertilizer (applied to agricultural land, 1974, 1982, 1992, 2002, 2012)	kg-N/km^2	Buffer mean	Alexander and Smith, 1990; Falcone, 2015; and Brakebill and Gronberg, 2017[15]
nnonf_YEAR	Nitrogen from non-farm fertilizer (applied to residential or public lands, 1974, 1982, 1992, 2002, 2012)	kg-N/km^2	Buffer mean	Alexander and Smith, 1990;[16] Falcone, 2015;[14] Brakebill and Gronberg, 2017[15]
nconf_YEAR	Nitrogen from applied manure (attributed to crops and pasture/hay land use, 1982, 1992, 2002, 2012)	kg-N/km^2	Buffer mean	Ruddy et al., 2006[17] and Falcone, 2015[14]
nucnf_YEAR	Nitrogen from grazing animals (attributed to crops, pasture/hay, and grazing lands, 1974, 1982, 1992, 2002, 2012)	kg-N/km^2	Buffer mean	Ruddy et al., 2006[17] and Falcone, 2015[14]
Mean_NfromFertilizer	Nitrogen from fertilizer (applied to agricultural land, 1992-2001)	kg-N/ha	Buffer mean	Nolan and Hitt, 2006[18]
Mean_NfromManure	Nitrogen from confined animal manure (1992-1997)	kg-N/ha	Buffer mean	Nolan and Hitt, 2006[18]

ModeledNO3_mean, ModeledNO3_median	Predicted nitrate concentration in groundwater	mg/L as N	Buffer mean or median	Ransom et al., 2022[19]
**Geology**				
ROCKTYPE	Main classes into which rocks are divided	Categorical	Point	Garrity and Soller, 2009[20]
Lithology	Major lithology	Categorical	Point	Kauffman et al., 2021[21]
SM_UNIT_NAME	Surface materials unit type, with some categories combined so at least 30 wells in each.	Categorical	Point	Soller et al., 2009[22]
SM_UNIT_THICK	Surface materials thickness (Patchy, < 100 ft, > 100 ft)	Categorical	Point	Soller et al., 2009[22]

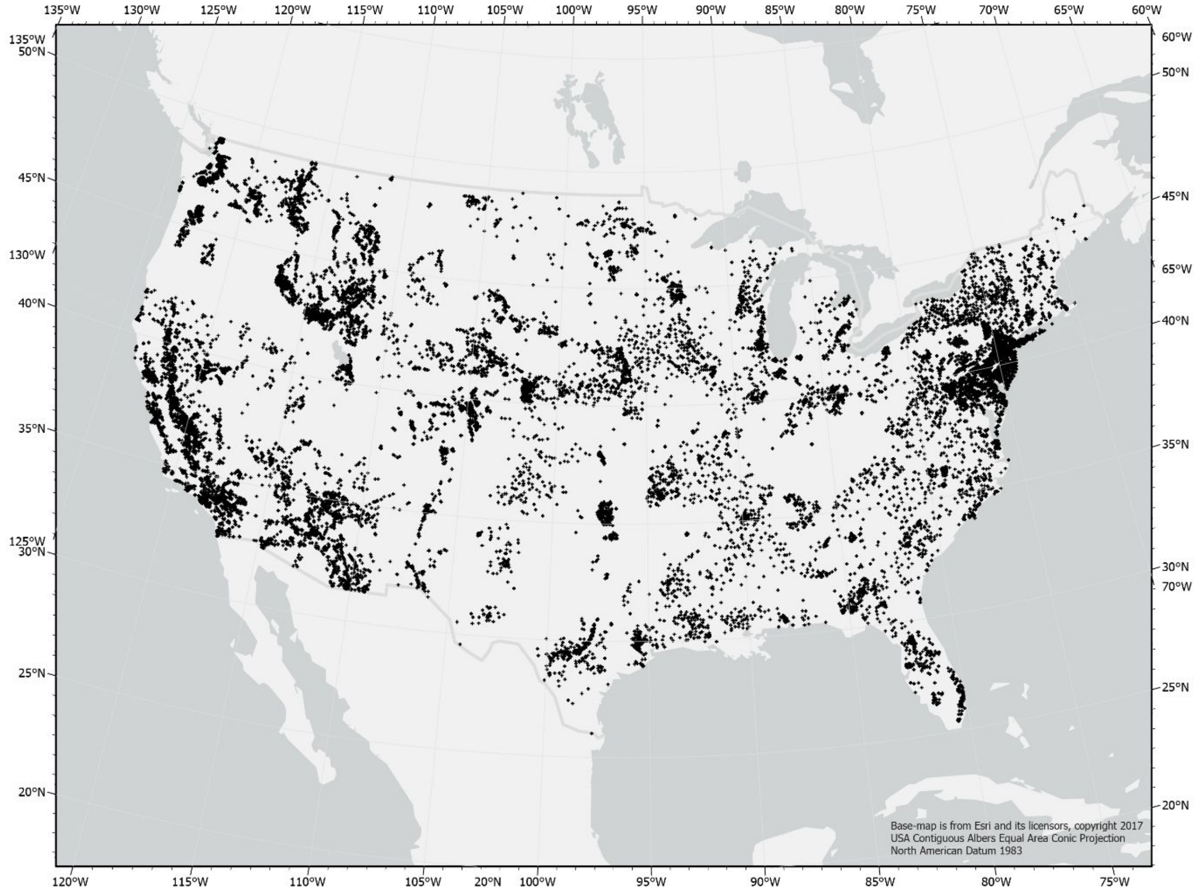

Figure SI-1. Sample locations for the data used to construct and test the random forest model that predicts the probability of oxic conditions in groundwater. See Wherry et al. (2023)[1] for more information.

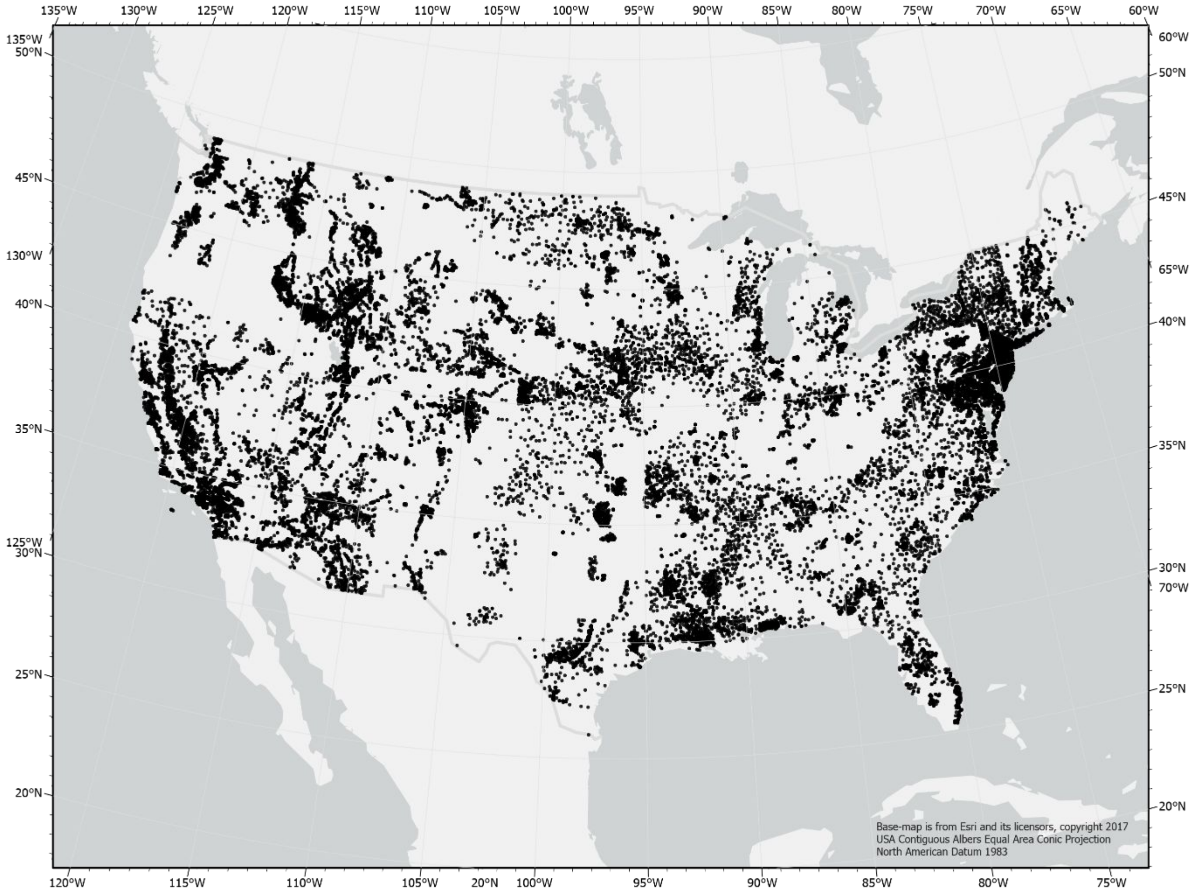

Figure SI-2. Sample locations for the data used to construct and test the random forest model that predicts the probability of elevated manganese concentrations in groundwater. See Wherry et al. (2023)[1] for more information.

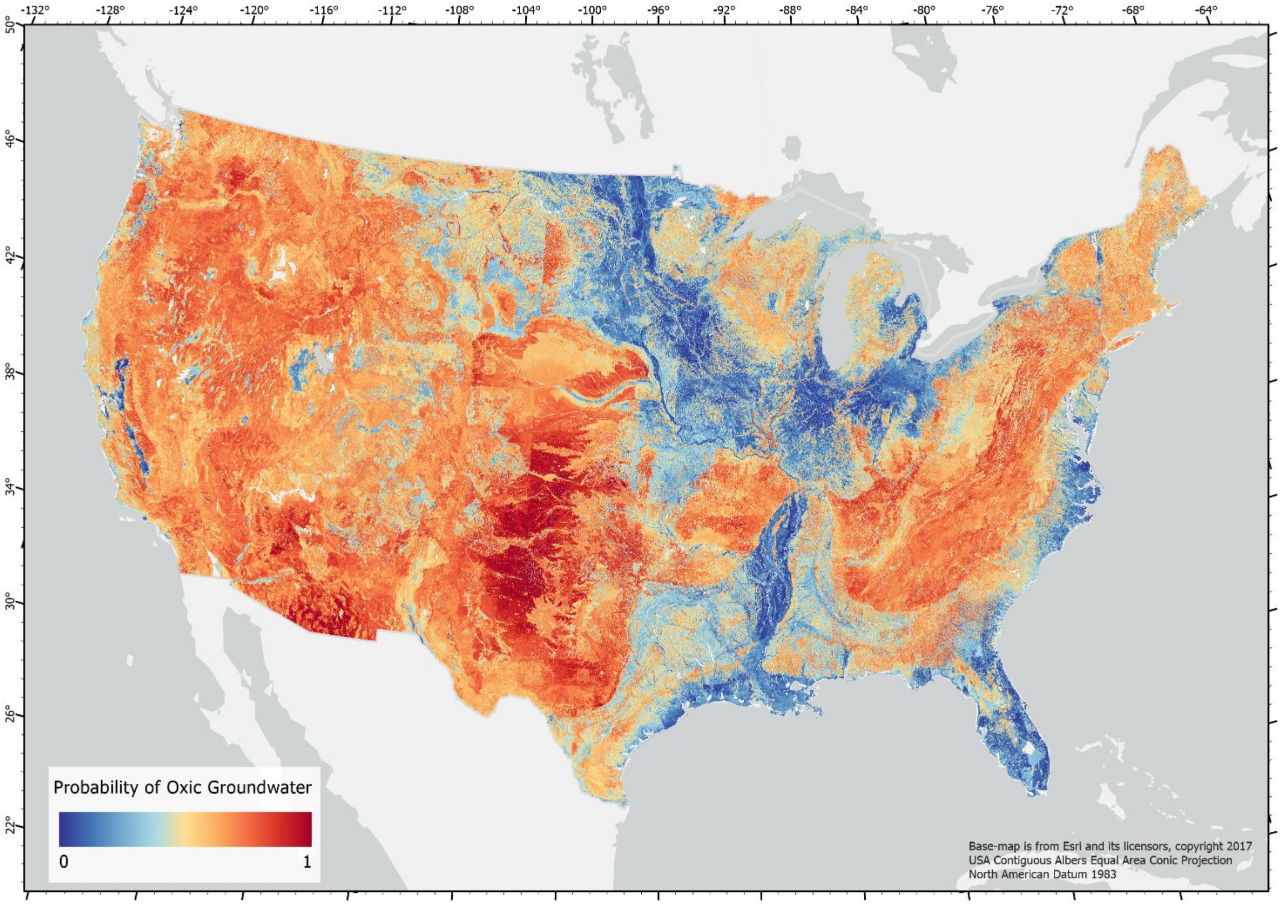

Figure SI-3. Map depicting the predicted probability of oxic conditions in groundwater that is 100 m below the water table.

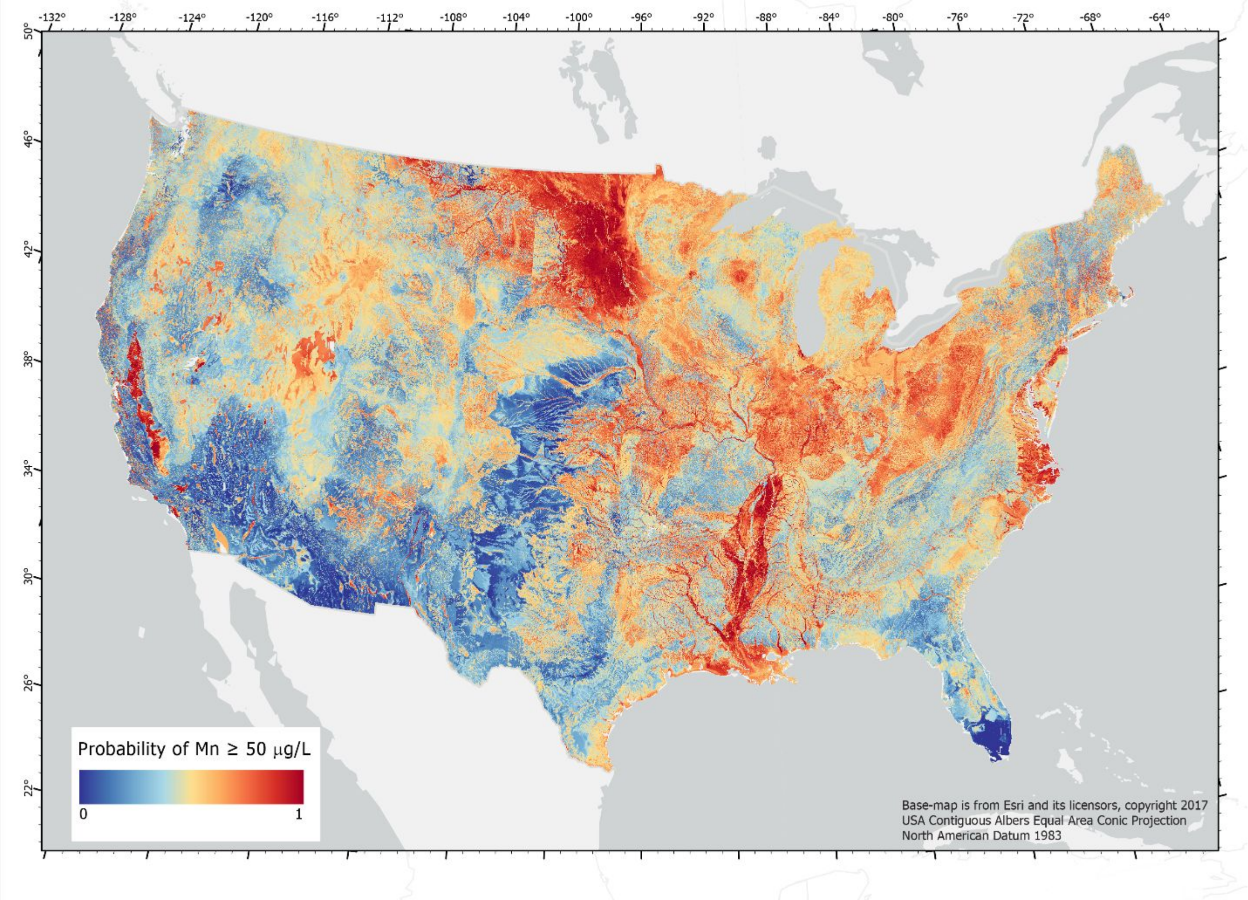

Figure SI-4. Map depicting the predicted probability of a manganese concentration ≥ 50 µg/L in groundwater that is 100 m below the water table.

References for Supporting Information

1. Wherry, S. A.; Tesoriero, A. J.; Dupuy, D. I., Input and results from a random forest classification (RFC) model that predicts redox conditions in groundwater in the contiguous United States. *U. S. Geological Survey Data Release, https://doi.org/10.5066/P9DVPJIX, accessed on February 2nd, 2024.* **2023**.
2. McMahon, P. B.; Belitz, K.; Reddy, J. E.; Johnson, T. D., Elevated manganese concentrations in United States groundwater, role of land surface–soil–aquifer connections. *Environmental Science & Technology* **2019,** *53*, (1), 29-38.
3. Zell, W. O.; Sanford, W. E., Calibrated simulation of the long-term average surficial groundwater system and derived spatial distributions of its characteristics for the contiguous United States. *Water Resour Res* **2020,** *56*, (8), e2019WR026724.
4. Belitz, K.; Moore, R. B.; Arnold, T. L.; Sharpe, J. B.; Starn, J. J., Multiorder hydrologic position in the conterminous United States: A set of metrics in support of groundwater mapping at regional and national scales. *Water Resour Res* **2019,** *55*, (12), 11188-11207.
5. Wolock, D. M., Base-flow index grid for the conterminous United States. *U.S. Geological Survey data release, https://doi.org/10.5066/P9MCTH3J, last accessed on January 12th, 2024.* **2003**.
6. Wolock, D. M., Estimated mean annual natural ground-water recharge in the conterminous United States. U.S. Geological Survey Data Reslease. Accessed from https://doi.org/10.5066/P9FSSVF3 on February 8th, 2024. **2003**.
7. Reitz, M.; Sanford, W. E.; Senay, G.; Cazenas, J., Annual estimates of recharge, quick-flow runoff, and ET for the contiguous U.S. using empirical regression equations. *J Am Water Resour As* **2017,** *53*, (4), 961-983.
8. Wolock, D. M., Saturation overland flow estimated by TOPMODEL for the conterminous United States, . *U.S. Geological Survey data release, https://doi.org/10.5066/P98MA1KO, last accessed on January 12th, 2024.* **2003**.
9. U.S. Geological Survey, USGS Small-scale Dataset - 1:1,000,000-Scale Hydrographic Geodatabase of the United States - Conterminous United States 201403 FileGDB 10.1. *U. S. Geological Survey Data Release, last accessed at https://www.sciencebase.gov/catalog/item/581d0551e4b08da350d5273e, on January 23rd, 2024.* **2014**.
10. PRISM Climate Group, United States Average Annual Precipitation and Temperature Data, 1981-2010 (800m; ASCII GRID). *Oregon State University, accessed from https://prism.oregonstate.edu/normals on August 11th, 2014.* **2014**.
11. Wolock, D. M., Hydrologic landscape regions of the United States. *U. S. Geological Survey Open-File Report 2003-145* **2003**.
12. Wieczorek, M. E., Area- and depth-weighted averages of selected SSURGO variables for the conterminous United States and District of Columbia. *U.S. Geological Survey Data Series 866* **2014**.
13. Smith, D. B.; Cannon, W. F.; Woodruff, L. G.; Solano, F.; Ellefsen, K. J., Geochemical and mineralogical maps for soils of the conterminous United States. *U. S. Geological Survey Open-File Report 2014-1082* **2014**, 399.
14. Falcone, J. A., U.S. conterminous wall-to-wall anthropogenic land use trends (NWALT), 1974–2012. *U.S. Geological Survey Data Series 948, last accessed from https://pubs.usgs.gov/publication/ds948 on January 24th, 2024.* **2015**, 45.
15. Brakebill, J. W.; Gronberg, J. M., County-Level Estimates of Nitrogen and Phosphorus from Commercial Fertilizer for the Conterminous United States, 1987-2012. *U.S. Geological Survey data release, last accessed from https://doi.org/10.5066/F7H41PKX on January 23rd, 2024.* **2017**.
16. Alexander, R. B.; Smith, R. A., County-level estimates of nitrogen and phosphorus fertilizer use in the United States, 1945 to 1985. *U.S. Geological Survey Open-File Report 90-130* **1990**.

17. Ruddy, B. C.; Lorenz, D. L.; Mueller, D. K., County-Level Estimates of Nutrient Inputs to the Land Surface of the Conterminous United States, 1982–2001. *U.S. Geological Survey Scientific Investigations Report 2006-5012* **2006**.
18. Nolan, B. T.; Hitt, K. J., Vulnerability of shallow groundwater and drinking-water wells to nitrate in the United States. *Environmental Science & Technology* **2006,** *40*, (24), 7834-7840.
19. Ransom, K. M.; Nolan, B. T.; Stackelberg, P. E.; Belitz, K.; Fram, M. S., Machine learning predictions of nitrate in groundwater used for drinking supply in the conterminous United States. *Sci Total Environ* **2022,** *807*, 151065.
20. Garrity, C. P.; Soller, D. R., Database of the geologic map of North America— Adapted from the map by J.C. Reed, Jr. and others (2005). *U.S. Geological Survey Data Series 425, retrieved from https://pubs.usgs.gov/ds/425/ on April 15th, 2018.* **2009**.
21. Kauffman, L. J.; Degnan, J. R.; Belitz, K.; Stackelberg, P. E.; Erickson, M. L., Data for depth of groundwater used for drinking-water supplies in the United States. *U.S. Geological Survey data release, https://doi.org/10.5066/P94640EM, accessed on August 9th, 2022.* **2021**.
22. Soller, D. R.; Reheis, M. C.; Garrity, C. P.; Van Sistine, D. R., Map database for surficial materials in the conterminous United States. *U.S. Geological Survey Data Series 425, accessed from https://pubs.usgs.gov/ds/425/ on April 15th, 2018.* **2009**.
